# Evaluating the Feasibility of Emotion Expressions in Avatars Created From Real Person Photos: Pilot Web-Based Survey of Virtual Reality Software

**DOI:** 10.2196/44632

**Published:** 2023-05-11

**Authors:** Anders Dechsling, Hugo Cogo-Moreira, Jonathan Spydevold Gangestad, Sandra Nettum Johannessen, Anders Nordahl-Hansen

**Affiliations:** 1 Department of Education, ICT and Learning Faculty of Teacher Education and Languages Østfold University College Halden Norway; 2 Department of Behavioral Sciences Oslo Metropolitan University Oslo Norway; 3 Department of Welfare, Management and Organisation Faculty of Health, Welfare and Organisation Østfold University College Halden Norway

**Keywords:** avatar, emotion recognition, emotion, face, facial expression, facial, images, real images, software, virtual reality

## Abstract

**Background:**

The availability and potential of virtual reality (VR) has led to an increase of its application. VR is suggested to be helpful in training elements of social competence but with an emphasis on interventions being tailored. Recognizing facial expressions is an important social skill and thus a target for training. Using VR in training these skills could have advantages over desktop alternatives. Children with autism, for instance, appear to prefer avatars over real images when assessing facial expressions. Available software provides the opportunity to transform profile pictures into avatars, thereby giving the possibility of tailoring according to an individual’s own environment. However, the emotions provided by such software should be validated before application.

**Objective:**

Our aim was to investigate whether available software is a quick, easy, and viable way of providing emotion expressions in avatars transformed from real images.

**Methods:**

A total of 401 participants from a general population completed a survey on the web containing 27 different images of avatars transformed, using a software, from real images. We calculated the reliability of each image and their level of difficulty using a structural equation modeling approach. We used Bayesian confirmatory factor analysis testing under a multidimensional first-order correlated factor structure where faces showing the same emotions represented a latent variable.

**Results:**

Few emotions were correctly perceived and rated as higher than other emotions. The factor loadings indicating the discrimination of the image were around 0.7, which means 49% shared variance with the latent factor that the face is linked with. The standardized thresholds indicating the difficulty level of the images are mostly around average, and the highest correlation is between faces showing happiness and anger.

**Conclusions:**

Only using a software to transform profile pictures to avatars is not sufficient to provide valid emotion expressions. Adjustments are needed to increase faces’ discrimination (eg, increasing reliabilities). The faces showed average levels of difficulty, meaning that they are neither very difficult nor very easy to perceive, which fits a general population. Adjustments should be made for specific populations and when applying this technology in clinical practice.

## Introduction

Perception and processing of facial expression and emotions through the use of images is a long-standing research field [1] and the use of facial emotion expression has become more common. Various sets of facial expressions have been developed for research purposes, deploying different facial expressions for different ethnicities [2]. The need for differing ethnicity samples of facial expressions follows the rationale that “within-group” processing of emotions is more readily available than “out-groups.” The use of facial expressions in web-based experimental research has also been on the rise, and databases such as the *Umeå University Database of Facial Expressions* [2].

Facial recognition and emotion training has for example been used in the treatment of anxiety and depression [3]. The potential for developing readily available databases for use with other groups with various diagnoses should be explored. However, some groups, such as many of those on the autism spectrum, are known to struggle with recognizing emotions in others [4]. For many people with autism, it would be beneficial to be able to recognize other people’s facial expression when maneuvering the society. This study aims to validate emotion expressions created by a software that uses real profile pictures that are transformed into avatars. One important reason to use such software is that children with autism seem to prefer avatars over real photos [5]. Interventions for children with autism should be individually tailored and the software could be a feasible way to quickly create the necessary material such as avatars made from people in the individual’s own environment. However, to make valid conclusions about the effects of an intervention, there is a need to validate the actual emotions expressed in the avatars. This proof-of-concept pilot survey therefore aims to investigate the feasibility in a general population first. With the knowledge on whether the emotions are correct or incorrect, it is possible to decide on the next step. Either proceed with investigating the facial emotion expression assessment of specific populations such as those on the autism spectrum or adjust the technology or use of it before proceeding further.

Autism spectrum disorders (autism from hereon) are characterized by challenges or differences in 2 main domains. The first domain relates to social interaction and communication. The American Psychiatric Association diagnostic manual indicates that the social communicational aspects are related to social-emotional reciprocity, for example differences in initiation and response in social interaction, nonverbal communicative behaviors, and developing and maintaining social relationships [6]. The second domain highlighted in the Diagnostic and Statistical Manual of Mental Disorders, Fifth Edition, is stereotypic and repetitive behaviors [6] and can be related to for instance fixed patterns of behavior, interests or routines, stereotyped motor movements, and hypo or hyperactivity related to sensory input. The prevalence of autism worldwide is estimated to be around 1% [7] and many individuals with autism need special education or other support systems [8]. It is important to acknowledge that there is a high heterogeneity between the individuals who fulfill the diagnostic criteria [9]. This means that the help and support individuals with autism might need should be tailored toward each person individually.

Young children diagnosed with autism tend to show more interest toward nonsocial stimuli than social stimuli [10]. The interest toward nonsocial stimuli might lead to children with autism missing out on social learning during early years, and thus hinder them in fulfilling potential desires for social interaction with peers at later stages in life. Social skills are thus considered an important target for interventions within autism research and clinical practice because of the possible difficulties highlighted as key domains to receive an autism diagnosis [11]. However, social interaction and communication are a highly complex domain consisting of a wide range of knowledge and skills of which the mastering criteria always depend on the context. One important area of social skills is to quickly recognize emotion expression in others and thereby behave and respond appropriately [12]. Facial expressions are therefore a subject in many social skill interventions and taught in most of the group social skill interventions [11]. Deficits in emotion recognition are associated with difficulties in social interaction [13] and as a predictor of difficulties in adaptive socialization [14]. The first step in responding to an emotion in another person’s facial expression is to identify the emotion expressed. Hence, there has been a focus to investigate [15] and teach recognition of facial expressions and emotions to individuals with autism [12,16]. These skills could be trained using immersive technology, thus reaping the benefits of the interest individuals with autism show towards computer-based environments [17].

Several researchers suggest that virtual reality (VR) technology could show promise in enhancing social skills [18,19]. VR is a term describing technology that displays potential real-world–like digital environments using visual and auditory stimuli through head-mounted displays (HMD), projectors or desktop or tablet devices with a possibility of interacting with that environment [20]. There are also different modalities of HMDs and projector setups. For instance, VR HMD consists of wearable goggles with inbuilt screens that give the user a feeling of being completely surrounded by the virtual environment and various versions of VR HMD provide various levels of digital interaction possibilities. Augmented reality (AR) is technology wherein digital components or images are superimposed on or blended with the real-world environment [21], often viewed through a mobile phone or tablet screen, or AR HMD (most often referred to as AR glasses or smart glasses). VR projector setups could range from Kinect technology using a projector and a screen in combination with motion sensors, to a full cave automatic virtual environment that consists of projectors and screens surrounding the user [22]. The potential uses of VR have led to an increase of its application in educational and special education settings [23], and in particular the amount of research on the use of VR for individuals diagnosed with autism [24,25].

Importantly, VR has shown to be an acceptable tool for individuals with autism in general [24] and for individuals with autism in need for more comprehensive support [26]. More than 1 in 10 studies on autism and social skills in VR/AR target emotion recognition behaviors [27]. Farashi et al [28] identified, in their systematic review and meta-analysis, a positive influence from VR or computerized training in emotion recognition by individuals with autism. After including 23 studies that focused on autism and VR or computerized training programs for emotion recognition, they calculated an overall effect size that was relatively large (*d*=.69)—considering the autism context [29]. However, the results obtained from Farashi et al [28] should be evaluated with caution since it is a quite heterogenic sample of studies. Some studies have also used AR in the form of smart glasses in training emotion recognition [30,31].

Children with autism show a preference toward the digital avatars as opposed to a human assistant [5]. We therefore consider social cues provided by avatars more in line with stakeholder preference in the early stages of skill training. This might be one of the main arguments for using avatars. Additionally, most of the studies included in Farashi et al [28] used facial avatars and they suggest that this could have positive effects for individuals with autism. Pino et al [32] concluded that children with autism experience less difficulties with recognizing emotions expressed by avatars as opposed to real images, and through eye tracking it was discovered that avatar faces were more explored than real faces. However, creating avatars with ecological valid expressions remains a possible challenge. Emotion expressions in general or specific populations do not necessarily differ per se. Therefore, the faces can be used interchangeably for the various population although there are numerous variables that can affect the recognition of emotions [33] such as for example ethnicity [2]. There are several “picture banks” developed, but many receive criticism related to the number of images or their representativeness [2]. In many cases, there is a need for individually adjusted exercises and therefore facial expressions from the persons in the target individual’s actual network could be more helpful and useful than unknown persons. A software allowing photos of people to create emotion expressions in avatars could solve several issues related to, for instance, sufficient material or representativeness. Consequently, a possible pitfall might be the validity of the expressions, and how to create such faces. In Pino et al [32], their expressions were validated through 2 psychologists and 20 typically developing children. In contrast, Tsai et al [34] applied virtual technology in emotion recognition but do only state to have validated the emotions in beforehand without stating how.

In sum, emotion recognition is a frequent target in social skills interventions using immersive technology, especially for individuals with autism [27]. Avatars could contribute to positive effects [28] since they appear as the most preferred [5] and explored, as well as perceived as less difficult to assess [32]. When considering the claim and call for tailored interventions, and the research showing that children with autism might prefer avatars as opposed to real images, we here investigate whether an available software that can transform profile pictures into avatars is a quick, easy, and viable way of providing various emotion expressions in avatars created from the individual’s actual surroundings (caregivers, teachers, peers, etc). That is, we investigate features that easily allow for manipulating the emotions expressed by the avatars and test whether the program makes expressions that are perceived correctly, according to the program settings, by a general and unspecific population. As a starting point and for piloting reasons, we use an unspecific population (ie, general population sample), meaning that we do not exclude any specific population such as for example an autism population, since we believe that an unspecific population will be more representative for a general assessment of the emotion expressions. This evaluation could determine whether such software could be used at later stages when training the specific skill of emotion recognition for individuals in various specific populations including those with autism.

Therefore, the overall research objective is to investigate whether an emotion expression software provides valid emotion expression when tested in a general population? More specifically our research questions are as follows:

- Research question 1: Are each emotion displayed by the avatars perceived correctly by the participants?
- Research question 2: What is the discrimination level between the images?
- Research question 3: What are the levels of difficulty in the images?

## Methods

### Participants

All 401 participants who completed (data on noncompleters are not applicable) the survey were recruited through social media platforms with an open invitation and link to respond to the survey. The survey was open for 8 weeks. Participants were asked to report their gender, whereas 86.8% (348/401) reported to be female, 13% (52/401) males, and 1 individual responded as “other.” The geographical origin of the participants was Scandinavia (382/401, 95.3%), rest of Europe (14/401, 3.5%), and spread around the world (5/401, 1.6%). The majority of participants were aged between 36 and 55 years (see Multimedia Appendix 1 for age distribution). The survey was piloted by 3 experts in the field and adjustments to the length were made prior to the publication of the survey.

### Ethical Consideration

A formal ethical review from an ethical committee was not required for this study because no identifiable or health-related information was gathered from the participants. This has been reviewed and confirmed by the responsible faculty dean in line with the institutional guidelines. Confidentiality principles were safeguarded through the officially approved web-based survey tool *Nettskjema* that ensures proper data protection services (nettskjema@usit.uio.no). No identifying information (eg, IP address) was collected. All participants were provided with information on data protection and that by proceeding with the survey they made their voluntary informed consent, of which they could withdraw by exiting the survey, as recommended in the general guidelines of the Norwegian National Research Ethics Committees. No compensation was given for participation.

### Apparatus and Stimuli

The survey was made using *Nettskjema*, a survey solution developed and hosted by the University of Oslo (nettskjema@usit.uio.no), which also ensures proper data protection services. This survey tool presents a fixed layout with possibilities of conducting various types of surveys.

The pictures were made using the software Character Creator (developed and copyright by Reallusion Inc.)**.** This software has an artificial intelligence (AI) function that enables the user to upload any photograph of a person’s face, thereby transforming it to a 3D model of the person. This feature also has the ability to adjust and transform the face to make it unrecognizable in the case of a need for privacy protection. The software has a number of pregenerated facial expressions with an additional “expressiveness scale” that can be used to adjust the faces. Furthermore, the software enables the user to adjust facial features such as eyebrows, nose, and all other features.

A total of 36 pictures were designed, by VisuMedia, as a sample of avatars showing various emotions. The sample consisted of 4 different avatars with 9 different emotion expressions. The 9 emotions were the basic emotions Happy, Sad, Afraid, Angry, and the more “complex” Disgusted, Surprised, Interested, Bored, and Ashamed [12].

In developing the stimuli, real photos were uploaded to the headshot feature in the Character Creator 3 software. The photos were transformed to avatars using the Edit Facial feature. The preprogrammed and standard emotion settings were applied to these avatars with 100% on the expressiveness scale and exported as JPEG files.

### Survey

The survey is a systematic replication of Samuelsson et al [2], in terms of developing the questionnaire. The survey was accessed through a link that was distributed through social media platforms such as Facebook, open from April 28 to June 18, 2021. It was created using the service *Nettskjema*, which has some restrictions on layout and design that affected the presentation of the scales and photos in the survey.

The participant first received the instruction:

In this survey you will be presented with a number of images of faces with different emotion expressions. With every image there will be presented alternatives to different emotions and a scale from 0 to 9, where 0 indicates that you completely disagree and 9 that you completely agree. Place your answer on the scale based on your opinion. Your responses are completely anonymous and cannot be traced to you or your IP address.

After pressing the consent button another page appeared with the demographic questions. When pressing “next,” a new page appeared with the text: “You are now ready to start the survey. Note that only one image will appear in a single page even though the image is repeated on that same page. Press Next page to start.”

**Figure 1:**
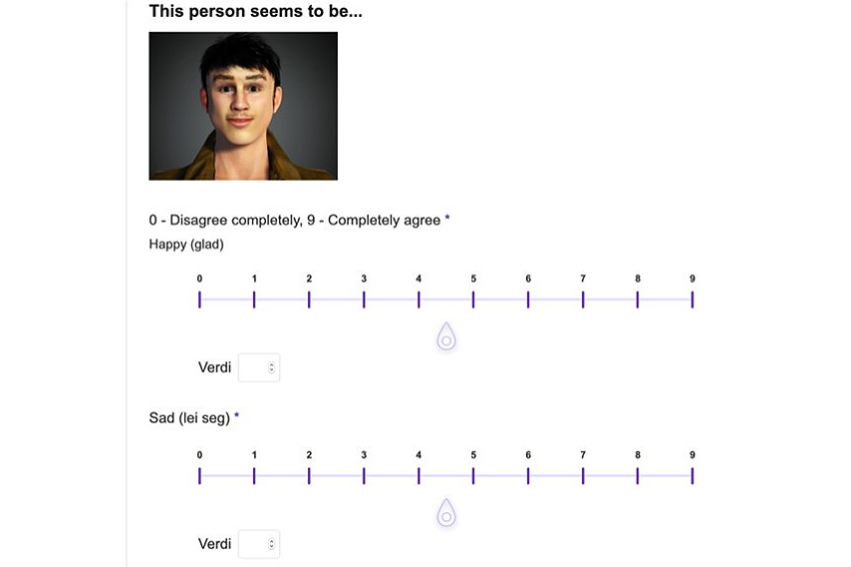
The survey page.

The participants were then presented with the text: “This person seems to be…,” the picture followed by all mentioned emotions. The participants were asked to rate each emotion to the same picture with a scale from 0 (disagree completely) and 9 (completely agree). See Figure 1 for an illustration.

**Figure 2:**
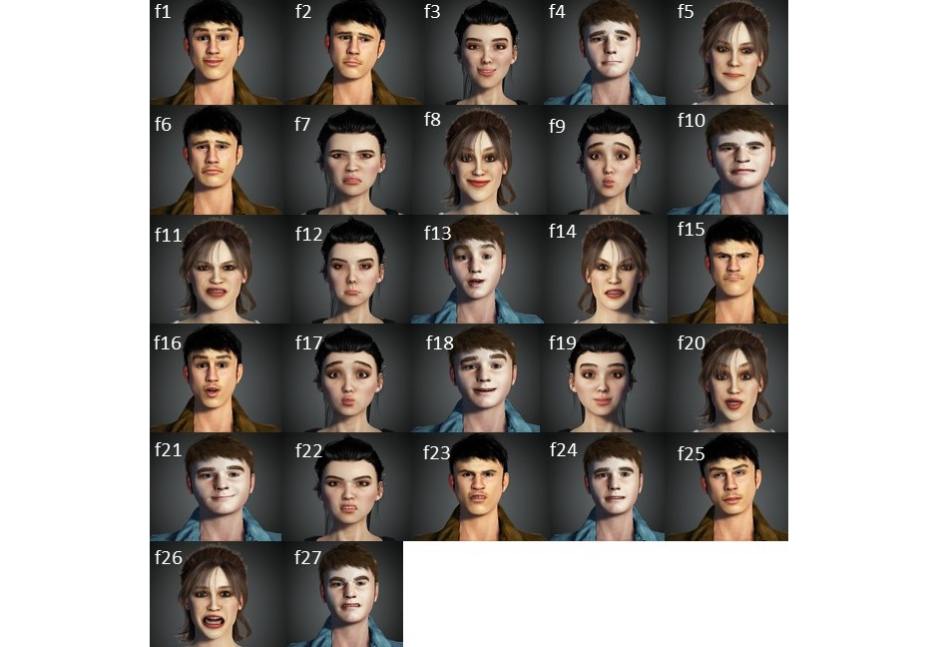
Images from the survey.

The pictures were presented serialized in separate pages. The order of presenting the pictures in the survey was determined using “List Randomizer” from the randomization service [35]. The last 9 pictures on the list were removed from the survey due to the length of the survey, meaning that 27 pictures were included in the survey (see Figure 2). All participants who completed the survey were presented with the pictures in the same order.

### Statistical Analysis

Based on Samuelsson et al [2], we

… [considered] an image to be correctly classified if the highest score was given to the emotion corresponding to the true emotion. For example, if the emotion ‘sad’ was scored seven and the other emotions between 0 and 6 points, then sad would be counted as the response (p. 3).

After this procedure, we obtained 27 dichotomous variables used as observed outcomes in a structural equation modeling approach. This approach calculates (1) the reliability of each image and (2) the level of difficulty (ie, the threshold) for each one of the face images. We used Bayesian confirmatory factor analysis testing, a multidimensional first-order correlated factor structure where faces showing the same emotion represented a latent variable. Therefore, we created 8 latent variables underlying the 27 observed variables. The Bayes estimator was used as it is compatible with such a high number of dimensionalities.

For all parameters (eg, factor loading and thresholds), we chose uninformative priors [36], assigned by default used in Mplus for dichotomous indicators. The priors are normally distributed with 0 mean and infinite variance.

Proportional scale reduction (PSR) was calculated to evaluate convergence. The Bayesian analysis used Markov chain Monte Carlo algorithms to iteratively obtain an approximation to the posterior distributions of the parameters. This approach was used to compare the variation of the parameters estimated in each iteration (called a chain). The PSR criterion essentially requires the between-chain variation to be smaller than the total of between- and within-chain variations. In terms of convergence, due to the complexity of the model, the minimum number of total iterations was 100,000, which included the discards.

The model under testing in this study was run until the chain goal reached a PSR value of 1.0. We used posterior predictive *P* values (PPP) to test the structural model for misspecifications. If the models fit the data well, the PPP would be close to 0.5. The corresponding 95% CI for the difference between the observed and the replicated chi-square value would range from a negative value to the same positive value and be centered on 0 [37,38].

## Results

### Research Question 1

Table 1 shows the percentage correctly perceived, meaning how often the emotion that models displayed was rated higher than all other emotions. The faces showing the highest percent of correctly perceived expressions were F4 (310/401, 77.3%) and F25 (296/401, 73.8%) and the lowest were F2 (12/401, 3%) and F8 (20/401, 5%).

**Table 1:** Percentage of correctly perceived emotions.

Image ID and outcomes			Proportion		Count
**F1**					
	Incorrect	0.895		359	
	Correct	0.105		42	
**F2**					
	Incorrect	0.970		389	
	Correct	0.030		12	
**F3**					
	Incorrect	0.401		161	
	Correct	0.599		240	
**F4**					
	Incorrect	0.227		91	
	Correct	0.773		310	
**F5**					
	Incorrect	0.915		367	
	Correct	0.085		34	
**F6**					
	Incorrect	0.394		158	
	Correct	0.606		243	
**F7**					
	Incorrect	0.481		193	
	Correct	0.519		208	
**F8**					
	Incorrect	0.950		381	
	Correct	0.050		20	
**F9**					
	Incorrect	0.454		182	
	Correct	0.546		219	
**F10**					
	Incorrect	0.870		349	
	Correct	0.130		52	
**F11**					
	Incorrect	0.698		280	
	Correct	0.302		121	
**F12**					
	Incorrect	0.756		303	
	Correct	0.244		98	
**F13**					
	Incorrect	0.349		140	
	Correct	0.651		261	
**F14**					
	Incorrect	0.406		163	
	Correct	0.594		238	
**F15**					
	Incorrect	0.347		139	
	Correct	0.653		262	
**F16**					
	Incorrect	0.229		92	
	Correct	0.771		309	
**F17**					
	Incorrect	0.446		179	
	Correct	0.554		222	
**F18**					
	Incorrect	0.908		364	
	Correct	0.092		37	
**F19**					
	Incorrect	0.791		317	
	Correct	0.209		84	
**F20**					
	Incorrect	0.414		166	
	Correct	0.586		235	
**F21**					
	Incorrect	0.683		274	
	Correct	0.317		127	
**F22**					
	Incorrect	0.703		282	
	Correct	0.297		119	
**F23**					
	Incorrect	0.778		312	
	Correct	0.222		89	
**F24**					
	Incorrect	0.516		207	
	Correct	0.484		194	
**F25**					
	Incorrect	0.262		105	
	Correct	0.738		296	
**F26**					
	Incorrect	0.416		167	
	Correct	0.584		234	
**F27**					
	Incorrect	0.559		224	
	Correct	0.441		177	

### Research Question 2

The 8-correlated factor model required 4700 iterations to meet the convergence criterion. A PPP of 0.182 was found for the model, and the 95% CI for the difference between the observed and replicated log-likelihoods ranged from –37.732 to 129.904, indicating an acceptable model. The factor loadings (Table 2) were all statistically significant (ie, the credibility interval does not cross 0); the highest factor loading (ie, reliability) was observed among face 13 (surprise; factor loading=0.733) and F8 (interested; factor loading=0.653). The lowest factor loading was F21 (happy; factor loading=0.323) and F15 (angry=0.336). By low reliability, it means that the expression cannot discriminate those participants who are able (and not) to identify the expression under evaluation correctly. The majority of the faces showed a reliability superior to 0.4 which is a common cutoff for a meaningful factor loading effect size [39]. Such a value represents that the face shares 16% of variance with the underlying factor).

**Figure 3:**
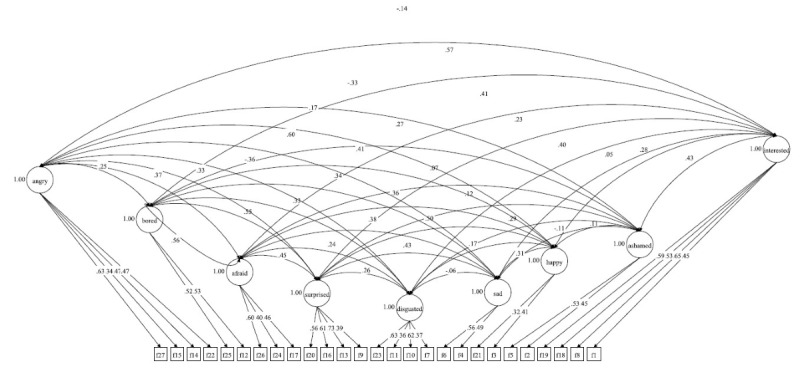
Intercorrelation between the 8-expression perception factor. Squares represent the 27 observed dichotomous indicators and the expression recognition factors ovals.

The highest correlation (Figure 3) was observed between happiness and anger (*r*=0.602), indicating that the more perception for anger someone has, the higher her or his perception of happiness will be. The lowest correlation was between sad and disgusted (*r*=–0.05), meaning that the recognition of both expressions is not correlated.

**Table 2:** Standardized factor loadings, posterior SD, 95% credibility interval, and significance (yes/no).

Emotion and faces			Factor loading		Posterior SD		95% credibility interval		Significance
**Interested**									
	F1	0.450		0.074		0.319-0.597		Yes	
	F8	0.653^a^		0.116		0.401-0.856		Yes	
	F18	0.527		0.111		0.309-0.737		Yes	
	F19	0.589		0.105		0.374-0.774		Yes	
**Ashamed**									
	F2	0.452		0.106		0.326-0.726		Yes	
	F5	0.525		0.176		0.116-0.840		Yes	
**Happy**									
	F3	0.412		0.062		0.310-0.548		Yes	
	F21	0.323		0.126		0.071-0.566		Yes	
**Sad**									
	F4	0.492		0.069		0.363-0.656		Yes	
	F6	0.559		0.122		0.309-0.796		Yes	
**Disgusted**									
	F7	0.366		0.057		0.278-0.500		Yes	
	F10	0.623^a^		0.120		0.357-0.831		Yes	
	F11	0.364		0.108		0.149-0.571		Yes	
	F23	0.628^a^		0.101		0.429-0.815		Yes	
**Surprised**									
	F9	0.394		0.051		0.303-0.509		Yes	
	F13	0.733^a^		0.074		0.573-0.870		Yes	
	F16	0.611		0.073		0.465-0.744		Yes	
	F20	0.565		0.078		0.407-0.714		Yes	
**Afraid**									
	F17	0.463		0.072		0.326-0.631		Yes	
	F24	0.401		0.093		0.214-0.571		Yes	
	F26	0.599		0.102		0.411-0.813		Yes	
**Bored**									
	F12	0.531		0.066		0.396-0.657		Yes	
	F25	0.516		0.093		0.325-0.680		Yes	
**Angry**									
	F22	0.466		0.064		0.364-0.617		Yes	
	F14	0.468		0.097		0.281-0.666		Yes	
	F15	0.336		0.094		0.147-0.517		Yes	
	F27	0.628^a^		0.090		0.442-0.808		Yes	

^a^The highest factor loadings.

### Research Question 3

Table 3 shows standardized thresholds (difficulty parameter), posterior SD, 95% credibility interval, and significance. F2 (ashamed, thresholds=1.881) might be seen as the more difficult face to be correctly rated, whereas the easiest was f16 (surprised; thresholds=–0.730).

**Table 3:** Standardized thresholds (difficulty parameter), posterior SD, 95% credibility interval, and significance (yes/no).

Face	Threshold	Posterior SD	95% credibility interval	Significance
F1	1.257	0.085	1.091 to 1.427	Yes
F2	1.881	0.120	1.661 to 2.133	Yes
F3	–0.249	0.063	–0.375 to –0.129	Yes
F4	–0.747	0.069	–0.879 to –0.609	Yes
F5	1.365	0.090	1.195 to 1.541	Yes
F6	–0.268	0.063	–0.390 to –0.144	Yes
F7	–0.047	0.063	–0.171 to 0.076	No
F8	1.638	0.102	1.438 to 1.844	Yes
F9	–0.115	0.063	–0.237 to 0.012	No
F10	1.121	0.079	0.965 to 1.278	Yes
F11	0.517	0.066	0.390 to 0.647	Yes
F12	0.692	0.069	0.555 to 0.830	Yes
F13	–0.378	0.064	–0.504 to –0.256	Yes
F14	–0.236	0.063	–0.358 to –0.112	Yes
F15	–0.390	0.064	–0.517 to –0.265	Yes
F16	–0.730	0.069	–0.864 to –0.596	Yes
F17	–0.131	0.063	–0.254 to –0.006	Yes
F18	1.323	0.087	1.153 to 1.497	Yes
F19	0.800	0.069	0.669 to 0.939	Yes
F20	–0.214	0.063	–0.338 to –0.090	Yes
F21	0.477	0.066	0.345 to 0.603	Yes
F22	0.535	0.065	0.406 to 0.661	Yes
F23	0.767	0.068	0.633 to 0.902	Yes
F24	0.043	0.061	–0.078 to 0.162	No
F25	–0.636	0.071	–0.778 to –0.494	Yes
F26	–0.208	0.065	–0.336 to –0.079	Yes
F27	0.150	0.062	0.026 to 0.269	Yes

## Discussion

### Overview

The aim of this study was to evaluate the possibilities of using the face generator as a valid tool to quickly display various emotions transformed to avatars from actual profile pictures as a starting point. This would provide clinicians and trainers to quickly transform pictures of specific people into avatars. We tested the generated images in an unspecific population as an approach toward reducing potential confounding factors associated with deficits in evaluating emotion expressions.

In this proof of concept of using avatars directly transformed from profile pictures, we found, in contrast to Samuelsson et al [2], few “true” emotions in our sample. This indicates inaccuracies in perceiving the intended emotion from our avatars. Hence, in terms of our first research question, we cannot confirm that participants assessed the pictures according to the intended emotion. In investigating the second research question, we found that the factor loadings (ie, discrimination) are not high; only few faces showed factor loadings superior to 0.7, representing 49% of shared of variance with the latent factor where the face is linked on. The factor loadings indicate that the images endorse the minimum cutoff of 0.4 commonly used in the literature for a meaningful effect size, but this effect size should be considered with caution. 0.4 as a cutoff for factor loading represents 16% of common shared variance and consequently 84% is a measurement error. For clinical practice, we would suggest increasing the cutoff to closer to 0.7 in such a context, the images depicting higher factor loadings can be used for inspiration when trying to improve the discriminative features of other images. For example, F8 (interested) and F13 (surprised) showed the highest factor loadings even though they were considered more difficult (F8) and average difficulty (F13) to perceive according to the threshold values.

Threshold values closer to +3 would indicate that the emotion within the image was difficult to perceive, whereas the values closer to –3 would indicate an easier face. Threshold values close to 0 and between –1 and +1 are considered average. When we evaluate the standardized threshold for each image (ie, the levels of difficulty) to answer our third research question, it looks as most thresholds are around average. This indicates that the tasks are not too difficult nor easy, as only 5 images could be considered difficult. This could be seen as good enough for the general population, as in our sample, which would benefit from having the whole spectrum of difficulty levels in the test. However, in specific populations that one could argue might find evaluating emotion expression a bit more challenging (eg, autism), the thresholds should be lower and thus easier. This suggests that there is a need for easier items than the ones represented in our images.

Based on these results, we cannot confirm that plainly using software, exemplified with the Character Creator, is a valid approach on its own, given the lower effect size for the factor loading. Such usage might affect the validity evidence based on the response process of emotion recognition interventions that apply this approach. Furthermore, it is important to be aware of the possibility of whether interventions that might have used this kind of software have actually trained emotion recognition skills, or just tested the discriminative and difficulty of the presented facial expressions. That is, whether the interventionists are making fallacies about the effects on skills acquisition due to actually testing skills or whether they actually are just testing the ecological validity of the faces.

In our opinion, it is of great importance to individually evaluate people’s perceived emotions. The software used in our study seems highly feasible and easy to use, especially as a starting point in creating the avatars from profile pictures. Using the software settings alone does not seem like a quick fix, but the software allows you to adjust and make detailed modifications to the faces which makes it quite usable for specific settings. Furthermore, this feature might even enhance the possibility of morphing the stimuli and thus combine and use avatars and real faces interchangeably when suitable, as suggested by Pino et al [32]. When using avatars in research and clinical practice with individuals with autism or others that need tailored interventions, it is still important to validate each emotion before applying it in training. Generalization of skills is still a major issue in autism interventions and VR is proposed to mend on these issues; however, if the emotion expressed by avatars is not valid this could have detrimental effects rather than positive effects on generalization.

There are some limitations to our study. First, it is important to consider the fact that this survey was conducted with a sample from the general population meaning it includes a broad spectrum of people, in a nonrandom selection process. Therefore, cautious inference must be taken when discussing the possible implications of the results to other specific contexts (eg, participants with autism). There are many variables that can affect the recognition of emotions [2]. For example, the chosen profile pictures of the faces used in our study may have affected the results in some way. In addition, we have only used 1 software. The length of the survey may have led to fatigue that could affect the accuracy of the responses toward the end. There is a skewed distribution in terms of the gender of participants, as well as the cultural context. Therefore, our findings should be considered in this context. We have not controlled for any sequence effects since all images are presented in the same order for every participant due to the restrictions of the layout. As already mentioned, the PPP indicates that the factor model is acceptable but a better fit closer to .5 would be more preferable. Additionally, we suggest a higher cutoff score of the meaningful factor loading effect size as more preferable in a practical setting.

### Conclusion

Applying available software for using real images when creating avatars with various emotions is not as straightforward as it seems. The avatars did not display what referred to as “true” emotions when assessed by our participants. Therefore, we cannot confirm that using such software alone provides valid emotion expressions. Through our survey, and the avatars created by the software, we found that individual adjustments might be needed to increase the discrimination, as well as the level of difficulty for various populations. We therefore suggest evaluating the emotions for each use specifically before applying them in interventions to ensure the respective validity of the findings (ie, avoiding the fallacy of actually again evaluating the photos instead of training emotion recognition).

## Appendix

Multimedia Appendix 1 Distribution of participants in different age groups.

## Abbreviations

AI: artificial intelligence
AR: augmented reality
HMD: head-mounted display
PPP: posterior predictive *P* value
PSR: proportional scale reduction
VR: virtual reality

## Data Availability

The data sets generated and analyzed during this study are available from the corresponding author on reasonable request.
