# Supplementary figures and images for: Evaluating the Feasibility of Emotion Expressions in Avatars Created From Real Person Photos: Pilot Web-Based Survey of Virtual Reality Software
Source: JMIR Form Res. 2023 May 11;7:e44632. doi: 10.2196/44632 (PMC10214113; doi:10.2196/44632)

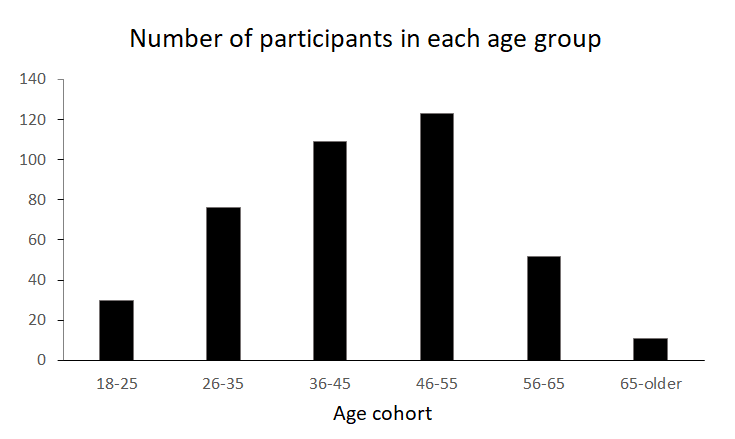

Supplement: Multimedia Appendix 1 [file formative_v7i1e44632_app1.png]
